# The Association between EEG Abnormality and Behavioral Disorder: Developmental Delay in Phenylketonuria

**DOI:** 10.5402/2012/976206

**Published:** 2012-03-29

**Authors:** Parvaneh Karimzadeh, Mohammad Reza Alaee, Hadi Zarafshan

**Affiliations:** ^1^Pediatric Neurology, Pediatric Neurology Research Center, Shahid Beheshti University of Medical Sciences (SBMU), Tehran, Iran; ^2^Pediatric Endocrinology, Pediatric Neurology Research Center, Shahid Beheshti University of Medical Sciences (SBMU), Tehran, Iran; ^3^Pediatric Neurology Research Center, Shahid Beheshti University of Medical Sciences (SBMU), Tehran, Iran

## Abstract

*Background.* Brain defect leading to developmental delay is one of the clinical manifestations of phenylketonuria. The aim of this study was to evaluate the association between EEG abnormality and developmental delay/behavioral disorders in phenylketonuria. *Patients and Methods*. 105 phenylketonuria patients, who were diagnosed through newborn screening tests or during follow-up evaluation, were enrolled. Patients who were seizure-free for at least six months before the study were included. The developmental score were evaluated by the ASQ questionnaire (age-stage questionnaire) and the test of child symptom inventory-4 (CSI-4), respectively. *Results*. 55 patients had a history of seizure more than 6 months before the study. Seventy had abnormal EEG (cases) and 35 had normal EEG (controls). There was no significant difference between mean phenylalanine levels in the abnormal and normal EEG groups at the time of diagnosis, after six months and at our evaluation. Distribution of DQ level in the abnormal and normal EEG groups revealed a significant difference. An abnormal EEG was associated with a higher percentage of low DQ levels. *Conclusion*. Paroxysmal epileptic discharges in PKU patients are important. Treatment of these EEG abnormalities may affect developmental scores or may lead to correction of some behavioral disorders in patients.

## 1. Introduction

Phenylketonuria is an autosomal recessive metabolic disease which may cause brain insult in the developing brain, consequently, leading to progressive neurodevelopmental delay.

In this genetic metabolic disorder, the hepatic enzyme, phenylalanine hydroxylase (PAH), is missing. This enzyme is necessary to break down amino acid phenylalanine to tyrosine. In deficiency of this enzyme, phenylpyruvate (phenylketone) may be detected in the urine [1].

Phenylketonuria is classified into classic phenylketonuria (PKU) which indicates phenylalanine (Phe) levels higher than 1200 *μ*mol/L and mild PKU in which Phe levels are between 600 *μ*mol/L and 1200 *μ*mol/L. In mild hyperphenylalaninemia, Phe level is higher than normal limits, but below 600 *μ*mol [1, 2].

In early infants with phenylketonuria, the serum phenylalanine level is in normal limits at birth, but begins to rise within the first few hours of life. Excessive phenylalanine is generally believed to be responsible for the brain insult leading to progressive mental retardation and seizure disorder. Without treatment, cognitive delay becomes evident within 6 months of age and is progressive [1, 2].

Although the principal biochemical defect is obvious in inborn errors of metabolism such as PKU and other similar disorders, the exact neurodysfunction defect is not clearly definite [1–3].

Hyperphenylalaninemia may be simply detected in the newborn screening test. Clinical manifestations in untreated infants with PKU include growth failure, microcephaly, seizures, dermatitis, and intellectual defect.

Remarkable clinical findings are light colored skin, hair and eyes, eczematous rash and behavioral disorders [1–4].

Different studies reported 25% generalized or partial seizures in phenylketonuria. Infantile spasms and myoclonic seizures are highly reported [4].

 Electroencephalogram (EEG) is a noninvasive method for cerebral investigation. It is useful for early detection of abnormal paroxysmal discharge in the brain. Long EEG recording can detect abnormal epileptic discharge with or without clinical seizure or convulsion.

EEG background activity is categorized into normal and abnormal background activity which is classified into type 1 and type 2 of continuous and hyperactive rapid tracing and in highly abnormal background activity, inactive, paroxysmal and low voltage plus theta activity.

In this study, we evaluated the association between EEG abnormality and developmental delay as well as behavioral disorders in patients diagnosed as phenylketonuria.

## 2. Materials and Methods

In this case-control study, 105 PKU patients (50 male, 55 female; mean age, 8.5 ± 6.2 years) who had been referred to the neurometabolic clinic of Mofid Children Hospital, Tehran, Iran from 2010 to 2011 were evaluated. These patients were diagnosed as PKU patients through newborn screening tests or later during follow-up evaluation for neurodevelopmental delay at the hospital.

The diagnosis of classic PKU was made on the basis of serum Phe level higher than 6 mg/dL in untreated infants, (We used mg/dL for measuring serum phe level.) Urine pterin analysis was done in order to exclude atypical PKU.

Before our study, the patients were matched according to sex, age, and the serum phenylalanine level.

An intensive medical and family history was recorded for all patients. Then all the patients were referred to the EEG laboratory for EEG recording (after sleep deprivation for 10 to 12 hours).

Pediatric neurologists took the history of seizures and all the patients who had recent seizures were excluded and only patients who were seizure-free for at least six months before the study were enrolled.

An informed written consent was obtained from parents or legal guardians.

Based on EEG recording, the patients were divided into two groups: the case group in which the patients had an abnormal EEG and the control group which consisted of those with normal EEGs.

 We evaluated the developmental score of the two groups with the ASQ questionnaire (age and stage questionnaire). After estimation of developmental score in cases and controls, we detected the developmental quotient (DQ) of patients using the below equation:

(1) $$\begin{array}{c} DQ=\;\frac{\text{Developmental}\;\text{age}}{\text{Actual}\;\text{age}}\times \;100. \end{array}$$

We also used the test of child symptom inventory-4 (CSI-4) for evaluation of behavioral disorders in children. This behavior rating scale screens for DSM-IV emotional and behavioral disorders in children between 5 and 14 years of age.

The parents' checklist was used. Parent checklist contains 97 items in a four-point response scale. It can be scored to diagnostic model or symptom severity scores. Scoring is easy and quick.

We evaluated Generalized Anxiety Disorder, Attention Deficit, Hyperactivity, Attention Deficit Hyperactivity Disorder, Oppositional Defiant Disorder, Compulsive Disorder, Obsession, and Autistic Behavioral Disorder in two groups.

We considered an EEG with normal background activity and mild epileptic discharge as mild abnormal EEG. EEGs with an abnormal background activity and definitely abnormal by showing paroxysmal epileptic discharge were considered as moderately abnormal. Severely abnormal EEGs were EEGs with continuous spikes and wave discharges or the hypsarrhythmia pattern.

Finally, the data were compared between the patients (cases and controls) and ANOVA and chi-square test were applied for statistical analysis. A *P* value less than 0.05 was considered significant.

## 3. Results

We evaluated 105 patients (50 boys, 55 girls) with the diagnosis of PKU. The mean age of the patients was 8.5 ± 6.2 years. Of these PKU patients, 52.3% (55 patients) had seizure (more than 6 months before the study) and 47.7% (50 patients) were clinically seizure-free.

The case group consisted of 70 patients (66.6%) with an abnormal EEG and the controls were 35 patients (33.4%) with a normal EEG. In the case group, 37 patients (52.8%) had a mild abnormal EEG, 10 patients (14.2%) had moderate abnormal EEG and 23 patients (32.8%) had severe abnormality in their EEG (as we considered in our evaluation and mentioned before).

In the case group, Phe levels were 14 mg/dL (mean, 21.4 ± 9.1 mg/dL) at diagnosis, 9 mg/dL (mean, 10.3 ± 1.6 mg/dL) after 6 months of diagnosis, and 4 mg/dL (mean, 7.31 ± 4.1 mg/dL) at the time of our evaluation. The serum phe levels in controls were 12 mg/dL (mean, 17.3 ± 6.1 mg/dL) at diagnosis and from 6 mg/dL (mean, 8.4 ± 2.1 mg/dL) after six months of treatment to 4 mg/dL (mean, 7.39 ± 3.6 mg/dL) at the time of our study.

There was no significant difference between mean phenylalanine levels in the abnormal and normal EEG groups at the time of diagnosis, after six months, and at our evaluation. Our group was matched according to serum Phe level (*P* > 0.05) (Table 1).

In the case group (patients with an abnormal EEG), 47% (33 patients out of 70 patients) had no clinical seizure. There was no significant difference between age in the two groups regarding DQ levels (above and below 80) (*P* = 0.2) and no significant difference in DQ levels between genders (*P* = 0.7).

Comparison between head circumference (HC) and the DQ level showed a significant difference between HC and DQ level in PKU patients (*P* = 0.04) (Table 2).

Distribution of DQ levels in the abnormal and normal EEG groups (cases and controls) revealed a significant difference; an abnormal EEG was associated with a higher percentage of low DQ levels (90% versus 57.1%) (*P* = 0.001) (Table 3).

Patients in the case group (abnormal EEG) according to severity of abnormality in EEG were categorized into three classes. Distribution of high and low DQ levels in these three subgroups showed a significant difference (*P* = 0.001) (Table 4). In other words, a severe abnormal EEG was associated with a higher percentage of low DQ levels.

In the behavioral-emotional scale evaluation, the frequency of attention deficit was 85.7% in the case group and 40% in the controls. Hyperactivity was detected in 71.4% and 28.5% of the cases and controls, respectively. 85.7% of the cases showed attention deficit hyperactivity disorders in comparison with 40% in the controls. Oppositional defiant disorders were reported 84.2% in the case group and 34.2% in the controls.

Generalized anxiety was observed in 74.2% and 42.8% in cases and controls, respectively. Autistic behavior was detected in 57.1% of the cases and 28.5% of the controls.

 Totally, the frequency of behavioral disorder was 85.7% in cases and 42.8% in control patients. There was a significant relationship between behavioral disorder and EEG abnormality in our study (*P* < 0.001) (Table 5).

## 4. Discussion

In neurometabolic diseases, neurodevelopmental delay, seizures and behavioral disturbances are the prominent presentations. According to our knowledge, different previous studies revealed the relationship between Phe level and EEG abnormality and also between Phe level and developmental delay, but there are limited studies about the relationship between EEG abnormality and developmental delay in PKU patients.

Donker et al. studied the effects of treatment of phenylalanine level (special diet) on the EEG of six PKU patients. Computerized EEG analysis showed activity appearance in the low frequency band (2–5 cycle/second), dominant rhythm frequency changes and synchronous degree changes between previous identified frequencies. This study described the association between phenylalanine level and EEG changes, when Phe level increased, the EEG changes intensified and in contrast when the Phe level decreased the EEG changes reversed, indicating that Phe or its metabolites lead to EEG abnormalities [5].

In our study, the case group consisted of 70 patients (66.6%) with abnormal EEGs. In our evaluation, we could not find any correlations between Phe level and EEG changes because we matched Phe level in the two groups. Between patients with abnormal EEGs (the cases) there was a significant relationship between EEG findings and Phe level (*P* < 0.001).

According to P. T. Gross et al. review, in EEG recording of PKU patients, about 45% of the patients had an abnormal EEG and nearly 30% had a normal EEG in the beginning which became abnormal later [6].

G. M. H. Abdel-Salam et al. study showed 25% of the patients had seizure, but more than 50% had an abnormal electroencephalogram, which means some PKU patients had an abnormal EEG without any clinical seizure [7].

In our study, 44% (28 patients) of the patients with abnormal EEGs (out of 63 patients in the case group) had no clinical seizure.

There is evidence that subclinical discharges can cause psychocognitive impairment and behavioral disturbance.

Karimzadeh and Tabarestani reviewing study reported negative effects of this epileptic form discharge on the choice reaction time, verbal and nonverbal communication and behavioral disorder [8].

Our study revealed 85.7% of the patients in the case group and 42.8% of the children in the control group had a pattern of behavioral disorders. These behavioral disorders were in accordance with their EEG abnormalities and behavioral disorders in phenylketonuria patients regardless of seizure and Phe level.

In Smith et al. study, the Rutter behavior questionnaire was used for evaluation of common abnormal behaviors in 544 patients with phenylketonuria (8-year-old children). Children had routine screening testing in the early months of screening program; anxiety, hyperactivity and less responsibility were detected. All patients received treatment before 4 months. They described that these abnormal behaviors may be the result of both psychologic stress and neurologic impairment [9]. They did not review these children's electroencephalograms.

 Rolle-Daya et al. also studied the EEG findings of 90 patients with classic PKU in one clinic. 73% of these patients who were diagnosed and were treated early had a normal EEG and 23% had mild abnormal background activity.

In patients diagnosed after 6 months of age, 31% had normal EEGs, 24% had abnormal background activity, and 45% had abnormal paroxysmal discharges. They did not assess the behavioral disorder or developmental EEG [10].

Thirty-four children with phenylketonuria with early treatment were studied by Pietz et al. They determined the prognostic value of age on the special diet in the first three months of life and IQ score and EEG were investigated [11].

Higher IQ scores were detected in patients with strict dietary control in comparison with lose control. EEGs of the 154 children were recorded. Alpha-activity development was normal while beta-activity was enhanced. Generalized slowing and paroxysmal activity (with or without spikes) were seen more frequently compared to the controls. EEG abnormality was increased independent of IQ development and EEG abnormality showed no relationship with the onset age or the quality of dietary treatment.

In our present study, we found a significant difference between DQ levels in the abnormal EEG group. 74.7% of the patients with abnormal EEG had a low DQ level; therefore, we had a significant relationship between abnormal EEG and the low DQ levels (Table 3). In addition, we found a significant correlation between severity of abnormal EEGs and the level of DQ in PKU patients (Table 4).

According to our study, (in PKU patients matched in Phe levels), there was a significant relationship between the abnormal EEGs and the low DQ level as well as the abnormal EEGs and behavioral disorders.

## 5. Conclusion

Based on our findings, the prevalence of seizure was less than EEG abnormality in PKU patients. There was a significant relevance between EEG abnormality and behavioral disorders and developmental score in phenylketonuria regardless of seizure or phenylalanine level.

The authors believe that physicians should pay attention to these paroxysmal epileptic discharges in PKU patients more than before and treatment of these EEG abnormalities may affect developmental scores or may lead to correction of some behavioral disorders in these patients.

## Figures and Tables

**Table 1:** Mean Phe level in case and control groups at the time of diagnosis, after six months and at our evaluation.

EEG Findings	Mean of Phe level (mg/dL) ± SD		
	At diagnosis	After 6 months of diagnosis	At our evaluation
Normal	17.3 ± 6.1	8.4 ± 2.1	7.39 ± 3.6
Abnormal	21.4 ± 9.1	10.3 ± 1.6	7.31 ± 4.1
*P* value	*P* > 0.05 (NS)	*P* > 0.05 (NS)	*P* > 0.05 (NS)

Phe = phenylalanine

NS = Non significant.

**Table 2:** Association between the mean head circumference and the level of DQ at birth and the time of our evaluation.

DQ level	Mean of HC (cm) ± SD	
	At birth	At our evaluation
<90	34.3 ± 1.2	49.2 ± 4.4
≥90	34.7 ± 1.5	48.3 ± 6.4
*P *value	*P* > 0.05 (NS)	*P* > 0.05 (NS)

DQ: Developmental Quotient

NS: Nonsignificant.

**Table 3:** Distribution of DQ level in cases and controls.

EEG Findings	DQ < 90	DQ > 90	Total	*P* value
	*N* (%)	*N* (%)		
(Controls) normal EEG	20 (57.1%)	15 (42.9%)	35	*P* = 0.003
(Cases) abnormal EEG	62 (88.5%)	8 (11.4%)	75	

**Table 4:** Distribution of DQ level based on type of EEG abnormality.

Type of abnormal EEG	DQ < 90	DQ > 90	Total
	*N* (%)	*N* (%)	
Mild	32 (45.7%)	5 (7.14%)	37 (52.8%)
Moderate	9 (12.8%)	1 (1.42%)	10 (14.3%)
Severe	21 (30%)	2 (2.86%)	23 (32.9%)
Total	62 (88.6%)	8 (11.4%)	70 (100%)

**Table 5:** Distribution of behavioral disorders in cases and controls.

Groups	Abnormal behavioral *N* (%)	Normal behavioral *N* (%)	Total	*P* value
Normal EEG (controls)	15 (42.8%)	20 (57.1%)	35	*P* < 0.001
Abnormal EEG (cases)	60 (85.7%)	10 (14.3%)	70	

## References

[1] Williams J, Berger D, Timothy G, Andrew’s DE (2011). *Disease of the Skin, Clinical Dermatology*.

[2] Nissenkorn A, Michelson M, Ben-Zeev B, Lerman-Sagie T (2001). Inborn errors of metabolism: a cause of abnormal brain development. *Neurology*.

[3] Kahler SG, Fahey MC (2003). Metabolic disorders and mental retardation. *American Journal of Medical Genetics*.

[4] Scriver CR, Kaufman S, Scriver CR, Sly WS, Childs B (2001). Hyper phenylalaninemia, phenylalanine hydroxylase deficiency. *The Metabolic and Molecular Bases of Inherited Disease*.

[5] Donker DN, Reits D, Van Sprang FJ, van Leeuwen WS, Wadman SK (1979). Computer analysis of the EEG as an aid in the evaluation of die tic treatment in phenyketonuria. *Electroencephalography and Clinical Neurophysiology*.

[6] Gross PT, Berlow S, Schuett VE, Fariello RG (1981). EEG in phenylketonuria. Attempt to establish clinical importance of EEG changes. *Archives of Neurology*.

[7] Abdel-Salam GMH, Abdel-Kader AA, Inical LE (2005). Electroencephalographic (EEG), Neuroradiological and Molecular (PKU) patients. *Egyptian JOurnal of Neurology, Psychiatry and Neurosurgery*.

[8] Karimzadeh P, Tabarestani S (2010). Promising medical treatment for childhood psycho-cognitive problems. *Neural Regeneration Research*.

[9] Smith I, Beasley MG, Wolff OH, Ades AE (1988). Behavior disturbance in 8-year-old children with early treated phenylketonuria. Report from the MCR/DHSS phenylketonuria register. *Journal of Pediatrics*.

[10] Rolle-Daya H, Pueschel SM, Lombroso CT (1975). Electroencephalographic findings in children with phenylketonuria. *American Journal of Diseases of Children*.

[11] Pietz J, Schmidt E, Matthis P, Kobialka B, Kutscha A, De Sonneville L (1993). EEGs in phenylketonuria I: follow up to adulthood; II: short-term diet-related changes in EEGs and cognitive function. *Developmental Medicine and Child Neurology*.

